# Imaging as a surrogate marker of drug efficacy in cardiovascular disease

**DOI:** 10.1136/heartjnl-2017-311213

**Published:** 2018-10-31

**Authors:** Jason M Tarkin, Marc R Dweck, James H F Rudd

**Affiliations:** 1 Division of Cardiovascular Medicine, University of Cambridge, Addenbrooke’s Hospital, Cambridge, UK; 2 National Heart & Lung Institute, Hammersmith Hospital, Imperial College London, London, UK; 3 Centre for Cardiovascular Science, University of Edinburgh, Little France Crescent, Edinburgh, UK

**Keywords:** cardiac imaging and diagnostics, positron emission tomographic (pet) imaging, coronary artery disease

**Learning objectives**

- Learn how imaging can be applied to gain early insights into drug efficacy and inform the design of phase III clinical outcome trials in cardiovascular disease.
- Understand which markers of atherosclerotic disease severity are most useful as imaging endpoints in drug intervention studies.
- Learn about the emerging role for molecular imaging of inflammation and disease activity in cardiovascular drug development.

## Introduction

Many cardiovascular drugs in the pipeline will fail to demonstrate a clear clinical benefit when evaluated in large-scale clinical outcome trials, which are costly, require lengthy follow-up and can potentially expose patients to unforeseen risks. There exists an enormous gap between early mechanistic studies demonstrating proof-of-principle drug efficacy in preclinical models and successful translation of these therapies into everyday clinical practice. To help overcome this challenge, cardiovascular imaging techniques can be applied to quantify early changes in disease severity owing to drug intervention, or lack thereof, with the aim of informing subsequent clinical outcome trials. This approach can be used to direct valuable resources towards development of drugs most likely to provide real clinical impact. The rationale here is that ‘surrogate’ imaging outcomes can be powered using far less subjects than clinical outcomes in drug trials, as each participant will contribute an imaging endpoint regardless of whether they then go on to develop a clinical event. In addition, drug efficacy can be more rapidly tested using imaging markers as there is no need to wait long periods of time for clinical outcomes to occur.

Imaging endpoints in clinical trials might also be used in the future to identify specific subgroups of patients who are more likely than others to respond to targeted pharmacotherapies in cardiovascular disease—the so-called precision medicine. Indeed, better methods are needed to identify those patients with cardiovascular disease who are most at risk of future or recurrent, clinical events despite secondary prevention. Many of these patients will have ‘residual’ on-treatment risk and could benefit from higher intensity lipid lowering or anti-inflammatory therapies currently under evaluation in atherosclerosis. Mechanisms underlying on-treatment residual risk are widely heterogeneous and patient specific, with different disease substrates (ie, thrombotic tendency, lipid accumulation and inflammation) contributing in varying degrees to an individual’s future cardiovascular risk. Consequently, applying targeted antiatherosclerotic drugs on top of standard therapies broadly in unselected patient populations is likely to produce at most a modest impact on clinical outcomes. This article will discuss the potential scope of imaging to improve drug efficacy testing of current and emerging disease-modifying therapies in atherosclerosis.

## Imaging endpoints

The ideal imaging endpoint for use in any cardiovascular drug trial should be easily measurable and highly reproducible, with sound biological rationale and strong prognostic link to important clinical outcomes. Importantly, an imaging biomarker should also reflect and track the mechanism of the tested drug. For example, measurement of plaque lipid content would be an appropriate biomarker for studying lipid-lowering therapies. The relative change in response to drug intervention for an ideal imaging endpoint should also be detected within a relatively short time-frame. Methods for quantification of atherosclerotic disease severity have been comprehensively reviewed elsewhere^1 2^; here we focus on their ability to predict clinical events.

### Validation of imaging for risk prediction

Among the most widely used imaging endpoints for cardiovascular drug trials are arterial inflammation, vascular intima media thickness (IMT), plaque burden (or atheroma volume) and plaque morphology.

Vascular inflammation is the earliest modifiable link between clinical cardiovascular risk factors and disease activity that can be detected using imaging. When imaged using ^18^F-fluorodeoxyglucose (FDG) positron emission tomography (PET), arterial inflammation can offer prognostic information beyond clinical risk factors, including Framingham risk score, with an increased HR of 2.9–4.7 for the highest risk groups in large retrospective analyses.^3 4^

IMT provides a measure of local atherosclerotic burden, including early subclinical disease, which has also been correlated with risk of future myocardial infarction (MI) and stroke.^5 6^ However, the link between vascular IMT and future cardiovascular risk remains unproven,^7^ and carotid IMT might represent vascular changes arising from arterial hypertension rather than a direct marker of atherosclerosis per se.^8^

Total plaque burden is in fact the strongest prognostic indicator that has been identified in large prospective imaging trials.^9–11^ While the presence of high-risk plaque features associated with the histological appearance of ‘vulnerable’ rupture-prone thin-cap fibroatheromas (TCFAs) are also predictors of major adverse cardiovascular events (MACE),^10–12^ it remains unclear whether identifying individual plaque characteristics is of incremental value to simpler assessments of plaque burden, as at the plaque level, this approach is limited by poor positive predictive value.^13^ Indeed, the vast majority of coronary artery TCFAs identified using virtual histology (VH)-intravascular ultrasound (IVUS) do not go on to cause clinical events because they either heal or rupture silently without clinical sequelae.^14^

New imaging techniques may offer opportunities to measure plaque lipid content, both invasively using near infrared spectroscopy and non-invasively using carotid MRI with T2 mapping.^15^ Quantification of pericoronary adipocyte content and inflammation could provide additional surrogate markers of cardiovascular risk for use in clinical drugs trials in the future.^16^

Degree of intraluminal stenosis, ischaemic burden and coronary artery calcification (CAC) are additional imaging markers that have been tested as surrogate endpoints in drug trials. While angiographic stenosis severity and functional ischaemia are among the most important factors used to guide everyday clinical management decisions, particularly when contemplating coronary revascularisation, they represent a late stage in the disease process that is not easily modifiable by drug intervention. Moreover, although there is a well-established association between ‘flow-limiting’ coronary disease and hard clinical outcomes, this relationship might not be causal. Indeed, reversal of coronary ischaemia with drug intervention and percutaneous coronary stenting in patients with stable angina does not appear to reduce rates of long-term MI or death.^17^ The presence of haemodynamically obstructive coronary stenoses might instead simply act as a surrogate of plaque burden. CAC scoring is another clinical risk stratification tool that provides an estimate of overall coronary atherosclerotic burden (including the burden of less stable plaques), with strong incremental link to clinical outcomes.^18^ However, the clinical significance of *change* in coronary calcification owing to drug intervention has yet to undergo specific validation as a prognostic biomarker and increases in coronary artery macrocalcification as observed in patients treated with statins might, in fact, be protective rather than harmful.^19^

### Other considerations

It is important to acknowledge that any perceived prognostic benefit of surrogate imaging markers, which has been inferred from observational studies, cannot stand alone for drug approval. Moreover, this approach does not account for the influence of confounding factors, including multiple drug effects and cannot replace the need for a prospective controlled clinical trial to test drug safety.^20^ Exposure to ionising radiation, additional risks associated with invasive imaging procedures and local accessibility to imaging technology are other factors to consider when choosing between surrogate imaging endpoints for cardiovascular drug trials. While plaque volume and composition can be more precisely quantified using invasive versus non-invasive coronary imaging, it is worth bearing in mind that there is also a high ~25% participant dropout in contemporary invasive imaging studies.^21^ In addition, the concept of an overall ‘barometer’ of disease severity that might be modifiable with drug intervention can be more readily attained using non-invasive than invasive imaging, for example, with PET or MRI, where the entire vascular bed can be imaged simultaneously.

## Use of imaging for testing drug efficacy in atherosclerosis

Here we discuss how various imaging biomarkers have been applied in clinical drug trials to study the efficacy of disease modifying therapies in atherosclerosis, including both long-established and newly tested lipid-lowering and anti-inflammatory agents.

### Lipid-lowering and other drugs affecting cholesterol

#### Statins

Statins reduce low-density lipoprotein cholesterol (LDL-c) through inhibition of β-Hydroxy β-methylglutaryl-CoA (HMG-CoA (HMG CoA)reductase and have been proven in landmark clinical trials to dramatically reduce the incidence of cardiovascular events in a range of individuals,^22^ with greater benefit seen for intensive versus moderate or low-dose therapy in patients with stable angina or previous MI.^23 24^ In fact, patients treated with statins who achieve LDL-c lowering of 2–3 mmol/L are expected to have a 40%–50% reduction in cardiovascular risk regardless of their baseline lipid profile.^25^ While the clinical benefits of statins have long been proven, contemporary imaging studies have nonetheless contributed important mechanistic insights revealing the multiple effects of statins on the arterial wall and atherosclerotic plaques. Collectively, these studies have demonstrated that treatment with statins can result in reduction of arterial inflammation, IMT, plaque volume and lipid content of the necrotic core, as well as a modest increase in angiographic luminal diameter and increased fibrous cap thickness and arterial macrocalcification contributing to plaque stability.

Dampening of arterial inflammation has been demonstrated in several studies of statins using ^18^F-FDG PET and ultrasmall superparamagnetic iron oxide (USPIO) nanoparticle-enhanced MRI. For example, ~11% reduction in the inflammatory ^18^F-FDG PET signal was observed in a study of statin-naïve patients treated with high-intensity atorvastatin after 12 weeks.^26^ Statin-induced dampening of arterial inflammation measured by ^18^F-FDG PET is associated with increases in high-density lipoprotein cholesterol (HDL-c) and the inflammatory biomarker matrix metalloproteinase-9.^27 28^ In the Atorvastatin Therapy: Effects on Reduction of Macrophage Activity study, significant reductions in carotid artery inflammation were also found using USPIO-enhanced MRI in patients treated with high-intensity statins.^29^

Regression of arterial IMT has been reliably observed in statin trials. In the Regression Growth Evaluation Statin study, treatment with pravastatin resulted in significant reduction in carotid and femoral IMT measured by B-mode ultrasound in patients with coronary artery disease.^30^ Similarly, the randomised placebo controlled Measuring Effects on Intima-Media Thickness: an Evaluation of Rosuvastatin study of 984 middle-aged individuals with low Framingham risk scores but evidence of subclinical atherosclerosis showed a reduction in ultrasound measured carotid IMT after treatment with rosuvastatin.^31^ Moreover, the beneficial effect of statins on IMT has also been demonstrated in several studies using MRI of the carotid arteries and the aorta, which also suggested that this drug therapy induces vascular remodelling by reducing atherosclerotic burden without affecting the lumen.^32 33^

Reduction in coronary artery plaque burden following treatment with statins has been demonstrated by numerous clinical studies using IVUS. Among the many longitudinal IVUS studies undertaken to investigate the effects of statins on coronary artery atherosclerosis are A Study to Evaluate the Effect of Rosuvastatin on Intravascular Ultrasound-Derived Coronary Atheroma Burden (ASTEROID),^34^ Reversal of Atherosclerosis with Aggressive Lipid Lowering,^35^ Early Statin Treatment in Patients With Acute Coronary Syndrome,^36^ Integrated Biomarkers and Imaging Study-4^37^ and Study of Coronary Atheroma by Intravascular Ultrasound: Effect of Rosuvastatin vs. Atorvastatin.^21^ Overall, these studies showed that high-intensity statin therapy is associated with significant reduction in percentage atheroma volume irrespective of baseline LDL-c or high-sensitivity C-reactive protein (hsCRP) levels. Moreover, a study including IVUS data from 4477 patients with stable angina demonstrated that individuals with high-risk plaques had accelerated progression of atheroma burden, which was modifiable in patients taking statins.^38^

**Figure 1:**
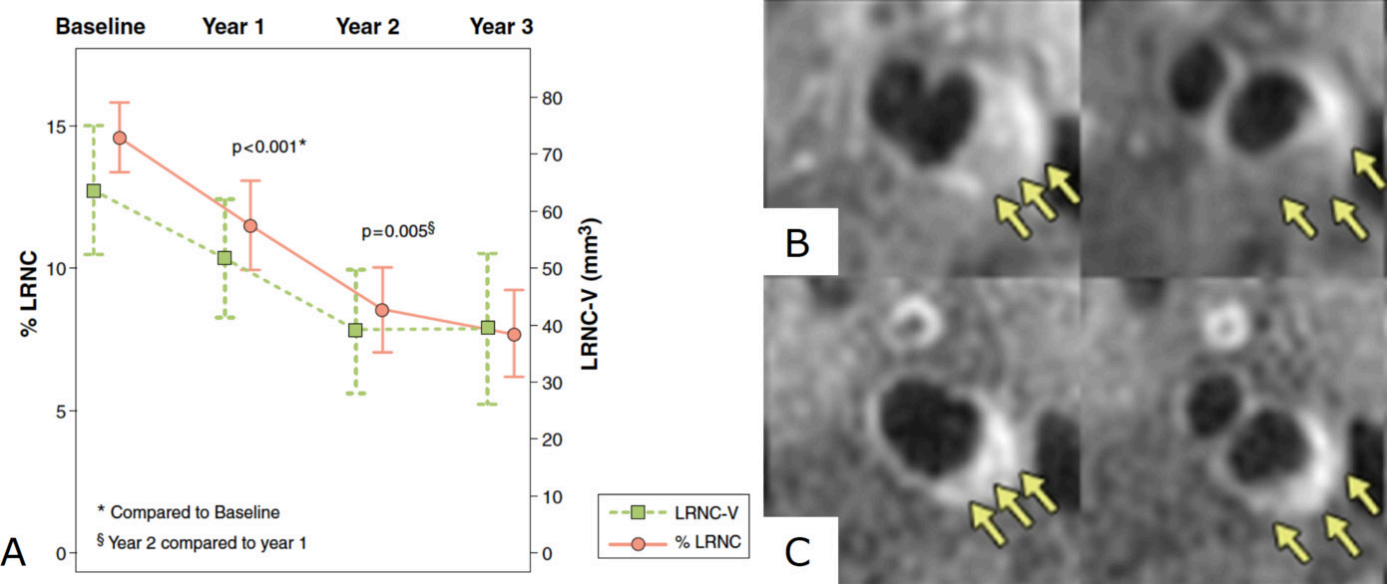
MRI of carotid lipid-rich necrotic core depletion after lipid-lowering therapy. (A) Graph showing significant regression of carotid artery lipid-rich necrotic core (LRNC) measured by serial MRI in a study of 33 individuals followed up over 3 years; representative MR images from this study at (B) baseline and (C) 3 years after lipid-lowering therapy demonstrating regression of the LRNC (arrows). Figure adapted from Zhao *et al*. *JACC Cardiovascular Imaging* 2011.^39^

Numerous imaging studies have shown that statins also induce favourable effects on plaque morphology. For example, in a study of 33 patients imaged using carotid MRI with follow-up over 3 years, significant reductions of lipid-rich necrotic core (figure 1), with accompanying increases in stabilising fibrous tissue, were observed in patients treated with intensive lipid-lowering including atorvastatin.^39^ Other studies using MRI have confirmed reductions in carotid plaque lipid-rich necrotic core and aortic plaque volume with rosuvastatin.^40 41^ High-intensity statin treatment was also associated with a reduction in coronary plaque necrotic core volume and number of TCFAs identified using VH-IVUS in several drug trials, including the Statin and Atheroma Vulnerability Evaluation study.^42 43^

**Figure 2:**
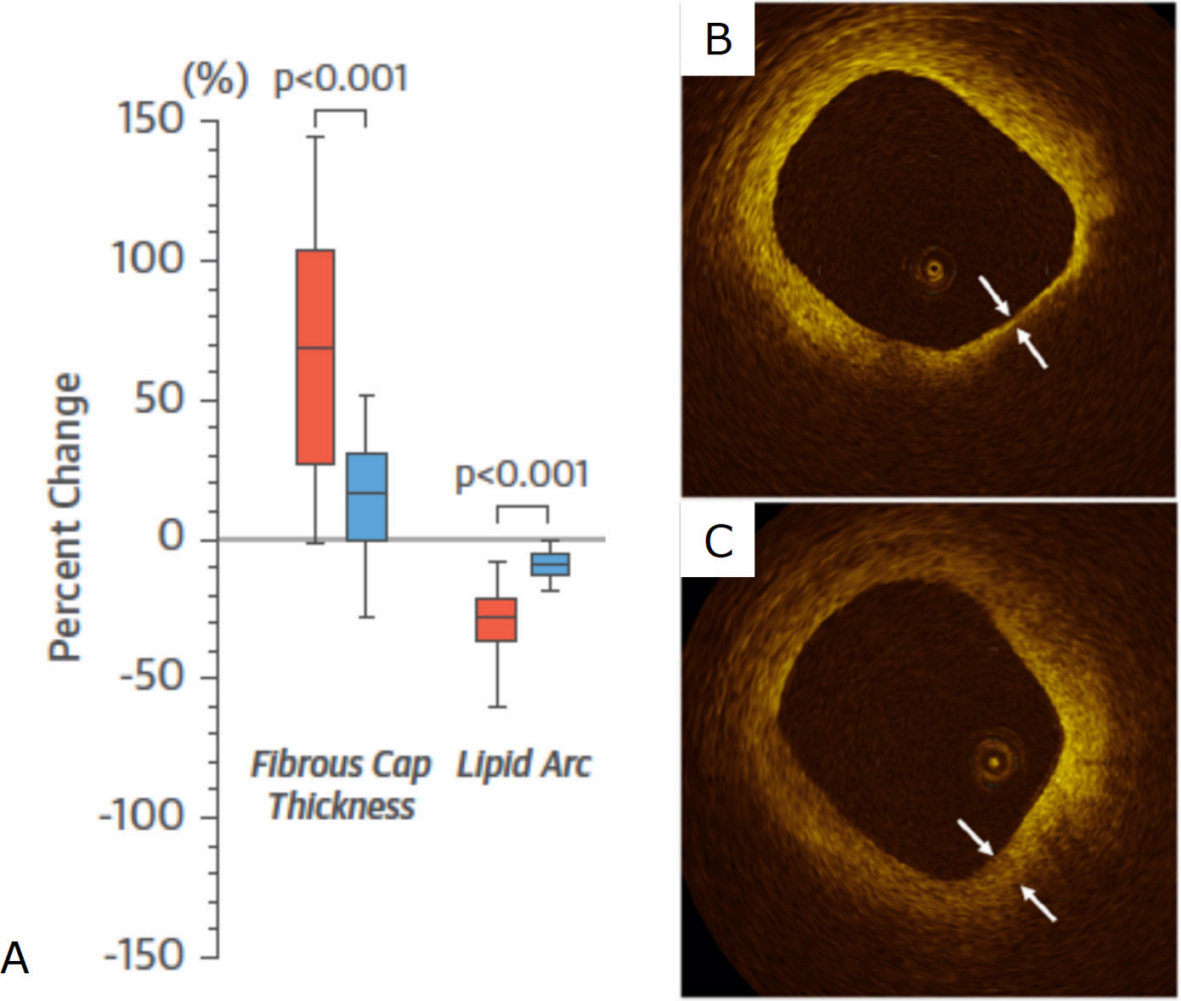
OCT imaging of fibrous cap thickening after statin treatment. (A) Graph showing per cent change in fibrous cap thickness and lipid arc measured by OCT in a study of 60 patients with unstable angina treated with atorvastatin 20 mg (red) or 5 mg (blue) for 12 months; representative OCT images from this study at (B) baseline and (C) 12 months showing increased fibrous cap thickness after treatment with atorvastatin. Figure adapted from Komukai *et al*. *J Am Coll Cardiol* 2014.^44^ OCT, optical coherence tomography.

Several trials have evaluated the effects of statins on fibrous cap thickness using optical coherence tomography (OCT). In the Effect of Atorvastatin on Fibrous Cap Thickness on Coronary Atherosclerotic Plaque as Assessed by Optical Coherence Tomography study, increased fibrous cap thickness measured by OCT occurred in correlation to reductions in LDL-c, inflammatory biomarkers and OCT-defined macrophage content (figure 2).^44^ In other OCT studies, patients treated with high-intensity statins had smaller lipid arcs and greater fibrous cap thickness compared with those on lower dose or no statins.^45 46^ Significant regression of coronary plaque lipid core content following treatment with rosuvastatin was also seen in the Reduction in Yellow Plaque by Aggressive Lipid lowering therapy (YELLOW) study using near infrared spectroscopy.^47^

Angiographic measurement of luminal narrowing has also been tested as a surrogate marker in drug trials evaluating the effects of statins. In the Multi-centre Anti-Atheroma Study (MAAS) trial, simvastatin resulted in a 2.6% increase in mean luminal diameter compared with placebo, with less patients showing angiographic disease progression in the treatment group.^48^ However, this surrogate marker was not correlated with extent of LDL-c change, and there was no difference in clinical outcomes between groups after 4 years. In a predefined substudy of the ASTEROID trial, a ~1% reduction in mean per cent diameter stenosis was observed using quantitative IVUS in patients treated with high-dose rosuvastatin, despite >50% reduction in LDL-c.^49^

**Figure 3:**
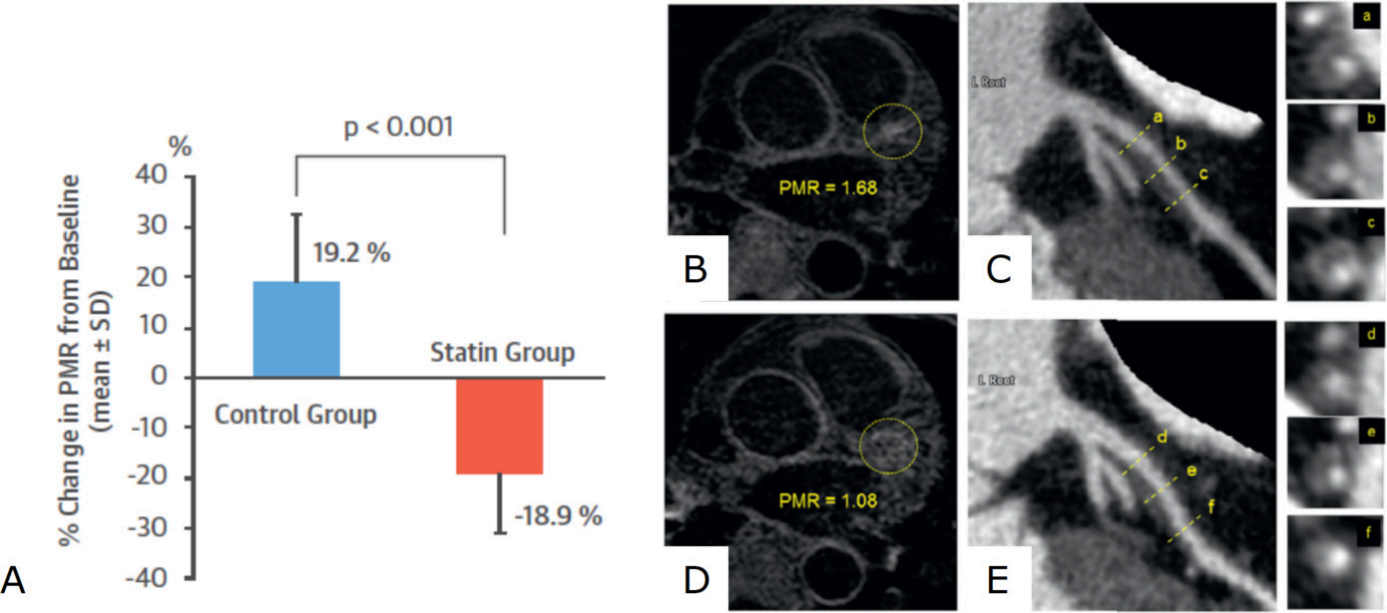
Effect of statins on T1-weighted MRI high-intensity plaques. (A) Graph showing significant reduction in T1-weighted MRI signal intensity in a study of 48 patients with coronary artery disease treated with high-intensity pivastatin compared with the propensity matched control group of patients with coronary disease not treated with statins; (**b**) representative image of a high-intensity proximal left anterior descending coronary artery plaque identified in this study using T1-weighted MRI, with (**d**) reduction in signal intensity after statin treatment; CTCA imaging of the same artery showing low-attenuation plaque and positive remodelling in the area of high-intensity on MRI (C) before and (E) after statin therapy showing reduction in plaque volume. Figure adapted from Noguchi *et al*. *J Am Coll Cardiol* 2015.^52^ CTCA, CT coronary angiography.

Studies using serial CT coronary angiography (CTCA) scanning have also shown that statins can slow progression of low attenuation and non-calcified plaques in patients with stable coronary disease.^50 51^ Indeed, the Attempts at Plaque Vulnerability Quantification with Magnetic Resonance Imaging Using Noncontrast T1-weighted Technique pilot study showed significant reduction in low attenuation plaque volume and percentage total atheroma volume measured by CTCA, as well as decreased plaque to myocardial signal intensity on T1-weighted MRI (a marker of high-risk plaque) following 12 months of treatment with statins (figure 3).^52^ However, in a randomised, double-blinded, multicentre trial including 471 patients with moderate CAC and without high-grade stenoses, statins were not able to attenuate progression of coronary artery macrocalcification.^53^ In fact, a paradoxical increase in dense calcific volume has been observed using CTCA in patients treated with high-intensity rosuvastatin after acute MI.^54^ This finding has been confirmed by a post hoc analysis of IVUS data from eight prospective randomised trials including 3495 patients, which showed increased plaque calcification following treatment with statins.^55^

#### Ezetimibe

As a second-line therapy for patients who are intolerant of statins or unable to achieve sufficient LDL-c reduction with statins alone, ezetimibe lowers LDL-c by reducing intestinal absorption of cholesterol. Ezetimibe has been shown to reduce cardiac events by a modest 2% compared with placebo when added to simvastatin in patients with acute coronary syndrome.^56^ This relatively small prognostic benefit compared with the large benefit afforded by statins might explain why imaging studies performed in patients treated with ezetimibe have shown somewhat mixed results.

**Figure 4:**
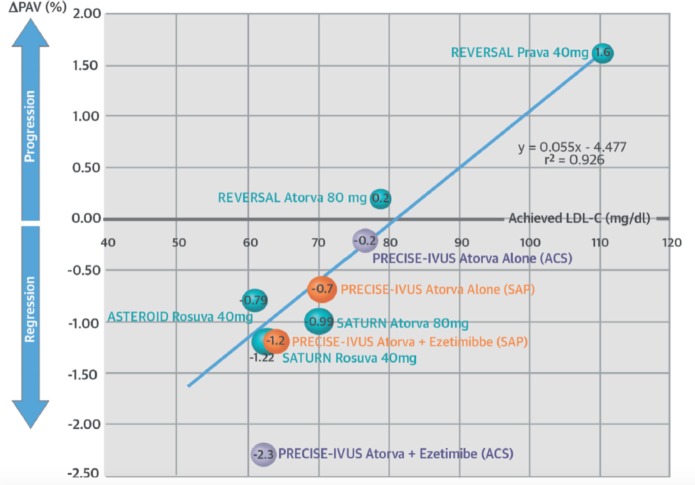
LDL cholesterol lowering versus IVUS-defined atheroma volume. Graph showing correlation between low-density lipoprotein (LDL) cholesterol and percentage atheroma volume measured in drug trials using intravascular ultrasound (IVUS). Figure from Tsujita *et al*, *J Am Coll Cardiol* 2015.^59^ ASTEROID, A Study to Evaluate the Effect of Rosuvastatin on Intravascular Ultrasound-Derived Coronary Atheroma Burden; LDL-c, low-density lipoprotein cholesterol; PRECISE-IVUS, Plaque Regression With Cholesterol Absorption Inhibitor or Synthesis Inhibitor Evaluated by Intravascular Ultrasound; REVERSAL, Reversal of Atherosclerosis with Aggressive Lipid Lowering; SATURN, Study of Coronary Atheroma by Intravascular Ultrasound: Effect of Rosuvastatin vs. Atorvastatin.

In the Ezetimibe and Simvastatin in Hypercholesterolemia Enhances Atherosclerosis Regression and Stop Atherosclerosis in Native Diabetics studies, ezetimibe did not significantly reduce carotid IMT when added to statins in patients with familial hypercholesterolaemia or diabetes mellitus, respectively, despite additional LDL-c lowering.^57 58^ While in the Plaque Regression With Cholesterol Absorption Inhibitor or Synthesis Inhibitor Evaluated by Intravascular Ultrasound study, the combination of high-dose atorvastatin plus ezetimibe resulted in greater regression in atheroma volume determined by IVUS than statins alone (figure 4), this difference failed to meet the predefined non-inferiority margin of 3%.^59^ In contrast, another study showed that the addition of ezetimibe to fluvastatin resulted in significantly reduced lipid arc and increased fibrous cap thickness by ~0.04 mm measured using OCT.^60^

Evidence from clinical trials using intravascular imaging also suggests that treatment with ezetimibe does not significantly alter plaque composition when added to statin therapy, despite additional reductions in LDL-c and plaque volume. Both the Virtual Histology of Atherosclerosis Regression During Atorvastatin and Ezetimibe Administration and Effect of Ezetimibe on Stabilization and Regression of Intracoronary Plaque studies showed no significant differences in plaque composition and stabilisation between patients randomised to statin plus ezetimibe versus standard therapy, or statin monotherapy, using serial IVUS imaging over the duration of these studies.^61 62^

#### Other drugs affecting cholesterol

Other cholesterol-modifying therapies tested using surrogate imaging markers include niacin and cholesteryl ester transfer protein (CETP) inhibitors (eg, dalcetrapib). While these studies mostly showed little or no beneficial effect on imaging endpoints, importantly, these findings predicted the negative results of the large-scale clinical outcome trials. The effects of the proprotein convertase subtilisin-kexin 9 (PCSK9) inhibitor evolocumab on coronary atherosclerosis has also been studied using imaging.

Among other effects on cholesterol, niacin acts primarily by increasing HDL-c ~20%. A meta-analysis of trials performed before statins became standard of care showed significant benefit on clinical outcomes and imaging biomarkers, including carotid IMT.^63^ High-dose modified release niacin also showed slight reductions in carotid artery wall area measured by MRI in statin-treated patients in the Oxford Niaspan study,^64^ as well as significant reduction in carotid IMT in patients without diabetes in the Arterial Biology for the Investigation of the Treatment Effects of Reducing Cholesterol)-2 (ARBITER) study, and in the ARBITER 6-HALTS (HDL and LDL treatment strategies) study with greater effect than ezetimibe.^65^ However, in the National Institute on Aging plaque study addition of niacin to statins did not reduce carotid wall volume assessed by MRI^66^; a finding that echoes contemporary clinical outcome data showing no added clinical benefit for niacin in patients treated with statins and ezetimibe.^67 68^

**Figure 5:**
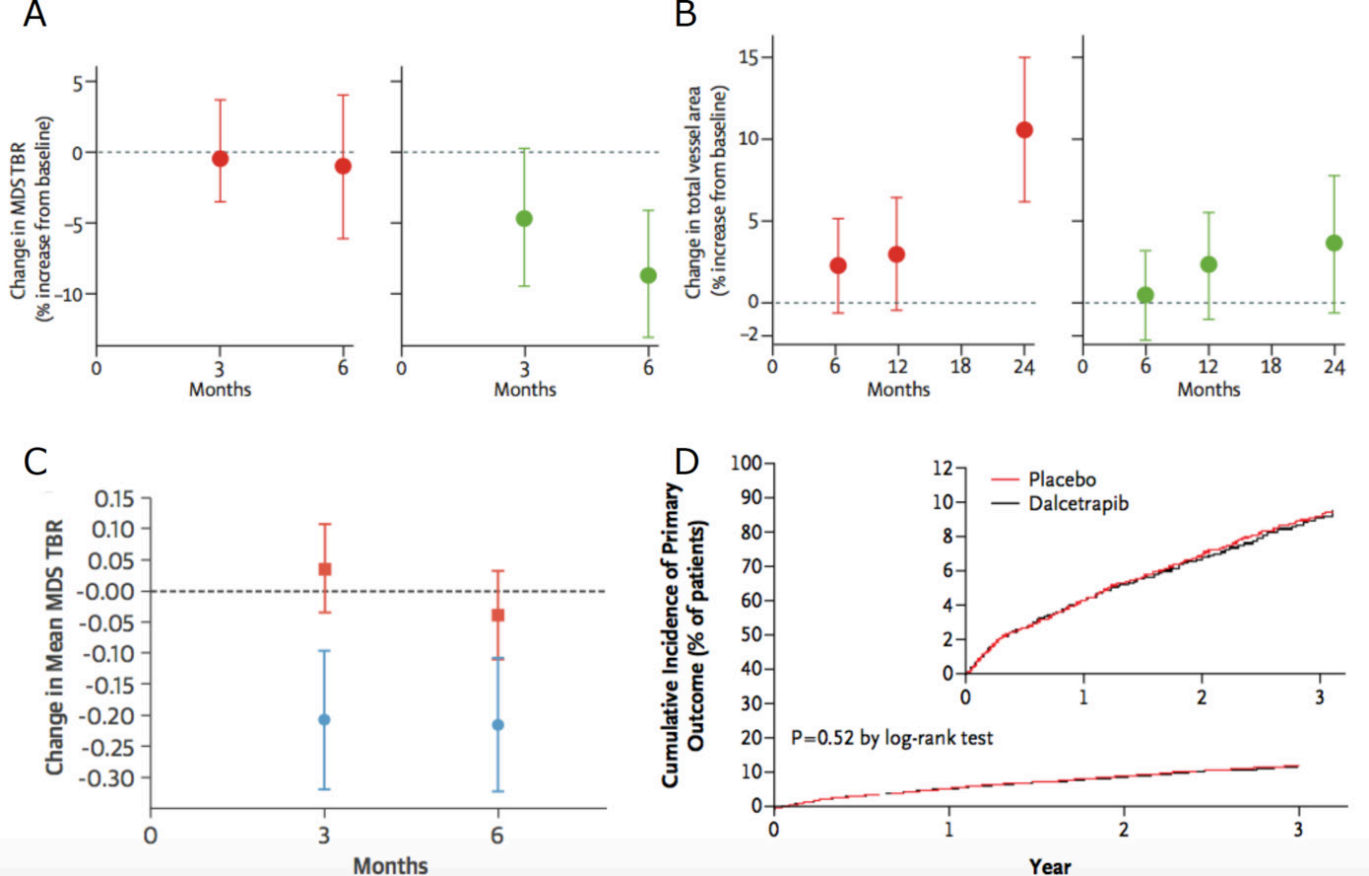
Use of surrogate imaging markers to evaluate dalcetrapib. Graphs showing nominal changes in (A) arterial ^18^F-FDG inflammatory signals (7% reduction, p=0.08) in the most inflamed regions and (B) carotid total vessel area (4 mm^2^ reduction; p=0.04) in a double-blind multicentre trial of 130 patients randomised to treatment with the cholesteryl ester transfer protein inhibitor dalcetrapib (green dots) versus placebo (red dots). In a substudy of the same trial, (C) the difference in carotid ^18^F-FDG signal intensity between dalcetrapib (blue dots) and placebo (red squares) was more apparent in patients without carotid calcification (p<0.001). However, lack of data showing a clear, consistent effect of dalcetrapib on these surrogate imaging markers predicted its inability to reduce recurrent cardiovascular events in patients with acute coronary syndrome in a large clinical outcome trial; (D) graph showing similar rates of cardiovascular events for dalcetrapib versus placebo in this study. Figure adapted from Fayad *et al*. *Lancet* 2011 (A and B);^73^ Joshi *et al*. *J Am Coll Cardiol* 2016 (C);^74^ Schwartz *et al*. *N Eng J Med* 2012 (D).^75^ MDS TBR, Most Diseased Segment Target-to-Background Ratio.

CETP inhibitors also act primarily by raising HDL-c. Torcetrapib is a CETP inhibitor that was withdrawn from the market due to concerns about off-target toxicity leading to raised systolic blood pressure and increased cardiovascular events.^69^ In the Investigation of Lipid Level Management Using Coronary Ultrasound to Assess Reduction in Coronary Atherosclerosis by CETP inhibition and HDL elevation study of 1188 patients with coronary artery disease imaged using serial IVUS, the addition of torcetrapib to atorvastatin did not significantly change the percent atheroma volume compared with atorvastatin monotherapy.^70^ The Rating Atherosclerotic Disease Change by Imaging with a New CETP Inhibitor-1 (RADIANCE-1) and RADIANCE-2 studies also showed that torcetrapib did not reduce progression of carotid IMT in patients with familial hypercholesterolaemia and mixed lipidaemia.^71 72^ Similarly, dalcetrapib showed only nominal reduction in arterial ^18^F-FDG PET signals and carotid wall area, and no change in vascular calcification, in the Safety and efficacy of dalcetrapib on atherosclerotic disease using novel non-invasive multimodality imaging (Dal-PLAQUE) study. Dalcetrapib also failed to improve clinical outcomes in the Effects of Dalcetrapib in Patients with a Recent Acute Coronary Syndrome (Dal-OUTCOMES) study, which was terminated early for futility (figure 5).^73–75^

**Figure 6:**
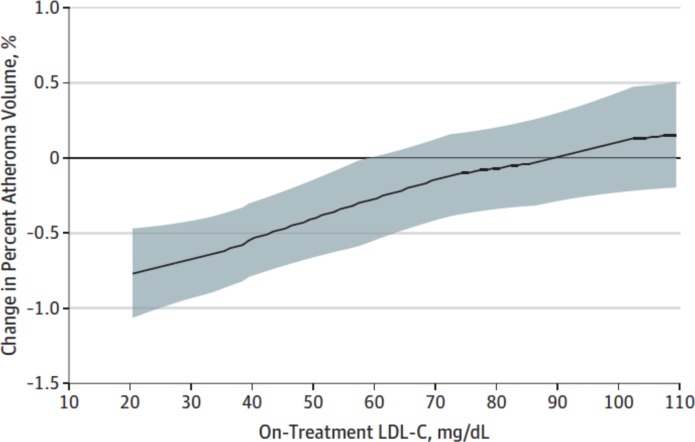
Effect of evolucumab on plaque volume versus low-density lipoprotein cholesterol (LDL-c). Graph showing the change in per cent atheroma volume measured by intravascular ultrasound in a study of evaluating the effects of aggressive LDL-c lowering with the proprotein convertase subtilisin-kexin 9 inhibitor evolucumab in statin-treated patients. Figure from Nicholls *et al. JAMA* 2016.^76^

PCSK9 inhibitors are human monoclonal antibodies that inactivate the enzyme PCSK9, preventing LDL-receptor degradation and reducing serum LDL-c by increasing its uptake into hepatocytes. In the randomised placebo-controlled Global Assessment of Plaque Regression with a PCSK9 Antibody as Measured by Intravascular Ultrasound (GLAGOV) trial that included data from 968 patients with coronary artery disease undergoing coronary angiography in 197 hospitals, the PCSK9 inhibitor evolocumab resulted in a modest ~1% reduction in plaque volume measured by IVUS (figure 6).^76^ Similarly, in a Cochrane review of clinical outcome trials evaluating PCSK9 inhibitors, a modest <1% reduction in cardiovascular events was demonstrated, despite a marked ~54% reduction in LDL-c compared with placebo.^77^ Results of the GLAGOV VH substudy, presented at the 2017 European Society of Cardiology Congress, showed that the addition of evolucumab to statin therapy did not significantly alter plaque composition measured by VH-IVUS (calcific, fibrofatty, fibrous or necrotic core volume), when compared with placebo.^78^

These modest effects coupled with little or no effect on all-cause mortality when applied to unselected patient cohorts,^77^ makes it difficult to justify the widespread use of expensive PCSK9 inhibitors. Indeed, the annual cost of a PCSK9 inhibitor (~$14 350) does not meet generally acceptable incremental cost-effectiveness thresholds.^79^ However, it is likely that higher risk cohorts would gain greater absolute clinical benefit, highlighting a potential role for imaging and other biomarkers to select those patients most likely to respond to treatment.^80^ Importantly, the Further Cardiovascular Outcomes Research with PCSK9 Inhibition in Subjects with Elevated Risk trial showed a significant reduction in cardiovascular events compared with placebo (9.8% vs 11.3%, p<0.001) in patients with atherosclerotic cardiovascular disease and raised LDL ≥1.8 mmol/L despite statin therapy.^81^ The ongoing A RaNdomized Double-blInd Placebo ConTrolled Study Characterizing THe Effects of PCSK9 Inhibition On Arterial Wall Inflammation in Patients With Elevated Lp(a), NCT02729025 study will determine whether evolucumab can reduce arterial inflammation measured by ^18^F-FDG PET in a cohort of patients with raised Lp(a) and LDL-c at baseline.

### Anti-inflammatory drugs

For decades now, we have known that atherosclerosis is an inflammatory condition and not merely a disease of lipid dysregulation. Local and systemic inflammatory networks fuel every stage of the disease process from initial lesion formation, to the progression, destabilisation, rupture and healing of advanced atherosclerotic plaques.^82^ Accordingly, a new wave of anti-inflammatory therapies are in development for the management of atherosclerosis targeted to a range proinflammatory pathways and mediators. In several instances, imaging has proven useful as an early marker of drug efficacy, or lack thereof, and again, it holds promise in identifying the patients most likely to benefit from these expensive or potentially toxic treatments.

#### Drugs used in systemic inflammatory diseases

Several disease-modifying and biological agents currently used for the treatment of chronic inflammatory diseases might be useful for treatment of atherosclerosis, including methotrexate, colchicine, tumour necrosis factor-α (TNFα) inhibitors and rituximab. Intriguingly, in a prospective controlled study of patients with severe psoriasis treated with anti-TNFα therapies or the interleukin (IL)-12/IL-13 inhibitor ustekinumab, these anti-inflammatory therapies halted progression of CAC score but not luminal narrowing assessed by CTCA over a 13-month period.^83^ Anti-TNFα therapy has also been shown to reduce arterial IMT in patients with psoriasis who did not have calcified atherosclerotic plaques^84^ and reduce aortic ^18^F-FDG inflammatory signals in patients with rheumatoid arthritis.^85^ In another study of 55 women with rheumatoid arthritis and without overt cardiovascular disease who were treated with rituximab, a monoclonal antibody to CD20 on B cells, a significant 9% reduction in carotid IMT was seen in those patients whose arthritis also responded to treatment.^86^ A prospective study of rituximab in patients with ST elevation MI is ongoing (NCT03072199).

#### Novel anti-inflammatory drugs for atherosclerosis

Among the many emerging therapies being evaluated for the treatment of atherosclerosis with anti-inflammatory actions include drugs targeted at p38 mitogen-activated protein kinase (MAPK), lipoprotein-associated phospholipase A_2 _(Lp-PLA_2_) and IL-1β. p38 MAPK is a proinflammatory stress-activated kinase present in macrophages, endothelial and myocardial cells, which among other mechanisms contributes to the amplification of the inflammatory cascade by promoting the release of cytokines, such as TNFα, IL-1 and IL-6. In a randomised placebo-controlled trial, the effects of the p38 MAPK inhibitor losmapimod on arterial inflammation were evaluated using ^18^F-FDG PET imaging in 99 patients with atherosclerosis who were also treated with statins.^87^ In this study, there was no significant difference detected in the primary endpoint of generalised vascular ^18^F-FDG uptake, although losmapimod did dampen ^18^F-FDG uptake in the most actively inflamed regions.^87^ In a subsequent clinical outcome trial including ~22 000 patients with acute MI, this drug did not significantly reduce the risk of major ischaemic cardiovascular events compared with placebo during the 12-week follow-up.^88^ In a study of another p38 MAPK inhibitor, BMS-582949, carotid and aortic ^18^F-FDG inflammatory signals were also not significantly lowered by this drug in patients with stable atherosclerosis receiving low-dose statins.^89^

Lipoprotein-associated phospholipase A 2 (Lp-PLA_2_) is another pharmacotherapeutic target that has been tested in cardiovascular disease. Increased Lp-PLA_2_ activity is associated with greater cardiovascular risk^90^ and, in preclinical studies, Lp-PLA_2_ inhibition has been shown to exert plaque stabilising effects mediated via anti-inflammatory actions on multiple genes associated with macrophage and T lymphocyte functioning.^91^ However, in a randomised placebo-controlled study of 83 patients with stable atherosclerosis imaged using ^18^F-FDG PET, the Lp-PLA_2_ inhibitor rilapladib showed no significant difference in the primary and secondary imaging end-points comparing vascular inflammation between treatment groups.^92^ Similarly, in another study, the Lp-PLA_2_ inhibitor darapladib failed to reduce coronary atheroma deformability (a marker of mechanical cap stress and plaque vulnerability) using IVUS palpography but did halt necrotic core expansion compared with placebo.^93^ Darapladib also did not significantly reduce the risk of MI, stroke or death in a randomised placebo-controlled trial of 15 828 patients with stable coronary disease followed up for 3.7 years,^94^ nor did it reduce major coronary events in a randomised, placebo-controlled trial of 13 026 patients followed up for 2.5 years after an acute coronary syndrome.^95^

Canakinumab is a human monoclonal antibody that inhibits IL-1β, a cytokine central to the acute inflammatory response that drives the classical IL-6 pathway. In a study of 189 individuals with atherosclerosis and type 2 diabetes mellitus or impaired glucose tolerance, there was no significant difference in mean carotid wall area on MRI observed after 12 months of drug treatment compared with placebo, despite measureable effects on hsCRP and IL-6.^96^ However, in a clinical outcome trial including 10 061 patients with previous history of MI and hsCRP >2 mg/L treated with canakinumab in addition to usual therapy, there was a significantly lower incidence of recurrent cardiovascular events compared with placebo.^97^ Further work is needed to fully evaluate the role of this, and other, anti-inflammatory drugs for the treatment of atherosclerosis.

## Conclusion

While we have many imaging strategies that can be applied as surrogate markers of drug efficacy, none of these methods can surpass the benchmark of a clinical outcome trial. However, imaging can be used to elucidate mechanisms of action and directly quantify specific drug effects on the arterial wall and atherosclerotic plaques. As we begin to see the clinical introduction of a range of novel antiatherosclerosis therapies to treat the many patients with so-called residual lipid or inflammatory burden, imaging can be used to help fast-track those drugs most likely to have a real clinical impact into large-scale phase III trials and to avoid wasting vast resources on drugs that have no measureable effect on any of the established markers of disease severity.

**Key messages**

- The use of imaging in clinical cardiovascular drug trials can impart valuable insights into underlying mechanisms of action and early evidence of drug efficacy to improve the efficiency of subsequent clinical outcome studies.
- Measurements of plaque burden are among the most useful surrogate imaging markers in cardiovascular disease, as this marker exhibits the strongest relationship with hard clinical outcomes.
- However, as modest changes in plaque burden occurring in response to lipid-lowering therapy do not match the large reductions in clinical outcomes observed in randomised trials, other mechanisms related to plaque composition and inflammation should also be considered for use as surrogate endpoints.
- Imaging endpoints cannot replace the need for large-scale clinical outcome studies to evaluate the true clinical value and safety of a new drug.
- Imaging studies have demonstrated that statins can dampen arterial inflammation, induce plaque regression and exert stabilising effects on plaque morphology and the degree of coronary macrocalcification.
- Imaging has also been used to test the effects of other cholesterol-modifying therapies, as well as anti-inflammatory therapies, on the arterial wall and atherosclerotic plaques.

**CME credits for Education in Heart**

Education in Heart articles are accredited for CME by various providers. To answer the accompanying multiple choice questions (MCQs) and obtain your credits, click on the ‘Take the Test’ link on the online version of the article. The MCQs are hosted on BMJ Learning. All users must complete a one-time registration on BMJ Learning and subsequently log in on every visit using their username and password to access modules and their CME record. Accreditation is only valid for 2 years from the date of publication. Printable CME certificates are available to users that achieve the minimum pass mark.
